# The Inferior Wedge Technique: A Novel Approach to Chest Masculinization with NAC Preservation

**DOI:** 10.1093/asjof/ojag138.016

**Published:** 2026-07-24

**Authors:** Erin Kelley, Andrea Battistini, Aneeq S Chaudhry, m Marco Ellis

**Affiliations:** Division of Plastic & Reconstructive Surgery, Northwestern University Feinberg School of Medicine, Chicago, IL, USA; Department of Plastic & Reconstructive Surgery, College of Medicine, The Ohio State University Wexner Medical Center, Columbus, OH, USA; Division of Plastic & Reconstructive Surgery, Northwestern University Feinberg School of Medicine, Chicago, IL, USA; Division of Plastic and Reconstructive Surgery, Florida International University Herbert Wertheim College of Medicine, Miami, FL, USA; Division of Plastic & Reconstructive Surgery, Northwestern University Feinberg School of Medicine, Chicago, IL, USA

## Abstract

**Goals/Purpose:**

Current techniques for chest masculinization include keyhole, periareolar, and double-incision mastectomies. Each technique is selected based on breast size, skin excess, and nipple position. The keyhole method is suited for small breasts with minimal laxity. The periareolar approach provides a concealed scar for small to moderate volumes but limited access to inferior tissue. The double-incision approach allows broad contouring for moderate to large breasts but creates longer scars and often reduces nipple sensation. However, patients with small to moderate breast volume, a long nipple-to-inframammary fold (IMF) distance, and inferior pole fullness or pseudoptosis are often not well served by these methods. This study aims to describe the inferior wedge technique, a novel approach to chest masculinization that improves lower pole contour, preserves nipple-areola complex (NAC) perfusion and sensation, and minimizes scar length to achieve enhanced aesthetic and sensory outcomes.

**Methods/Technique:**

A retrospective chart review was performed to identify all patients who underwent inferior wedge gender affirming mastectomy from 2024 - 2025. This technique was offered as a surgical option to patients who had small to moderate breast size as well as evidence of pseudoptosis on physical exam. The surgeries were all performed by a single surgeon. Data collected included age, body mass index (BMI), preoperative breast size, extent of vertical skin excision, NAC reduction, and postoperative outcomes.

The surgical technique shares some similarities to classic double-incision mastectomy. Patients were marked highlighting the native inframammary fold (IMF). After induction of general anesthesia and prophylactic intravenous antibiotics (cephalex 2 gm), incisional markings were confirmed. A horizontally oriented ellipse was designed consistent with the patient’s skin laxity. This ranged from 3-5 cm in vertical height. Approximately 250 mL of tumescent solution (1 L 0.9% normal saline, 500 mg lidocaine, 0.5 mg epinephrine, 12.5 mEq sodium bicarbinate) was injected to facilitate hydrodissection and hemostasis. The lower incision was made approximately 1 cm cephalad to the native IMF. Both incisions were made superior dissection along the breast capsule. The inferior flap was dissected towards the IMF. Both dissections continued to the chest wall fascia. The glandular tissue was excised in its entirety in a suprafascial fashion. Obliteration of the native IMF was performed with radial and circumferential capsulotomy. The superior mastectomy flap was mobilized along the insertion of the pectoralis fascia and axillar fascia to distribute tension. Optional areolar reduction involved the addition of two concentric incisions around the areola, deepithelialization between these incisions, and layered closure. Intraoperative pectoralis and serratus blocks were performed using lidocaine with epinephrine.

**Results/Complications:**

Eight patients underwent the inferior wedge gender-affirming mastectomy. The mean age was 25.3 years (range 19–31) and the mean BMI was 22.2 kg/m² (range 18.99–27.25). The cohort included two transmasculine and six nonbinary patients, all with small to moderate breast volume; six exhibited pseudoptosis and two had grade I ptosis. The average preoperative breast volume was approximately 300 g. The mean vertical skin excision measured 2.76 cm (range 1.2– 5.0 cm). Two patients underwent NAC reduction, with diameters reduced from 37 mm to 28 mm and from 40 mm to 30 mm. All patients achieved a symmetric, well-contoured masculine chest with preserved NAC perfusion and viability. There were no intraoperative or postoperative complications such as seroma, hematoma, or wound-healing issues. One patient required a minor secondary excision of residual subareolar tissue. All reported satisfaction with their aesthetic outcomes at an average six-month follow-up.

**Conclusion:**

The inferior wedge technique provides a novel alternative for chest masculinization in patients with smaller breasts and inferior pole fullness. It achieves a masculine contour with improved nipple positioning, optional nipple reduction, and a shorter IMF scar, while preserving NAC sensation and aesthetic quality. By maintaining NAC vascularity and allowing controlled repositioning, this approach provides a more natural appearance and reduces postoperative wound-healing concerns compared to free nipple grafting. Additionally, its design can be adapted to position the NAC centrally or retain partial areolar characteristics, offering flexibility for nonbinary patients seeking individualized aesthetic outcomes. As clinical experience with this technique expands, continued evaluation of aesthetic and sensory outcomes will further define its role within the spectrum of gender-affirming chest surgery.

